# Supplementary material for: Brainbuilder: a software pipeline for 3D reconstruction of cortical maps from multi-modal 2D data sets
Source: Commun Biol. 2025 Jul 7;8:1015. doi: 10.1038/s42003-025-08267-6 (PMC12234757; doi:10.1038/s42003-025-08267-6)
Supplement: Supplementary file 2 — Supplementary Information [file 42003_2025_8267_MOESM2_ESM.pdf]

5. Supplementary Information

5.1 BrainBuilder Usage

Figure 1. Illustrative example of BrainBuilder software usage

A) BrainBuilder python command

```
from brainbuilder.reconstruct import reconstruct

# launch reconstruction with br
reconstruct(
        'hemisphere_info.csv',
        'slab_info.csv',
        'section_info.csv',
        resolution_list=[4,3,2,1,0.5],
        '/path/to/output/'
)
```

B) User .csv inputs:

Hemispheric information in 'hemisphere_info.csv'

sub	hemisphere	struct_ref_vol	gm_surf	wm_surf
MR1	R	MR1_R_gm_srv.nii.gz	MR1_gray_surface_R_81920.surf.gii	MR1_white_surface_R_81920.surf.gii

Slab-level information in 'slab_info.csv'

sub	hemisphere	chunk	pixel_size_0	pixel_size_1	section_thickness	direction
MR1	R	1	0.027	0.020	0.02	rostral_to_caudal
MR1	R	2	0.038	0.029	0.02	rostral_to_caudal
MR1	R	3	0.040	0.030	0.02	rostral_to_caudal
MR1	R	4	0.039	0.029	0.02	caudal_to_rostral
MR1	R	5	0.039	0.029	0.02	caudal_to_rostral
MR1	R	6	0.030	0.023	0.02	caudal_to_rostral

Section-level information in 'section_info.csv'

raw	acquisition	hemisphere	sub	chunk	sample	conversion_factor
RG#hg#MR1s6#R#ampa#5679#04#L.TIF	ampa	R	MR1	6	1353	29.91
RG#hg#MR1s6#R#rx82#5700#21#L.TIF	rx82	R	MR1	6	467	41.15
RG#hg#MR1s6#R#sr95#5723#09#L.TIF	sr95	R	MR1	6	1091	36.42
RG#hg#MR1s6#R#ly34#5721#01#L.TIF	ly34	R	MR1	6	1491	121.40

A) sample piece of code illustrates the usage of BrainBuilder to perform the reconstruction of the human autoradiograph sections B) The user provides the essential information in simple .csv files. *Hemispheric information*: Each row specifies the information applicable to a single hemisphere, specifically the subject, hemisphere, the reference volume, and the associated cortical surfaces. *Slab information*: the rows specify the pixel sizes (in mm) and the direction of sectioning along the coronal axis. *Section information*: Each row specifies a single 2D image, its acquisition, the hemisphere and tissue chunk to which it belongs, the subject identification. The “sample” is its positioning along the coronal axis. The “conversion_factor” is an optional parameter that scales the images to convert the pixel intensities to a quantitative value, e.g., conversion of raw autoradiograph pixel intensities to fmol/mg protein.

5.2 Data Augmentation for segmentation U-Net

(1) Unique synthetic volumes were generated from the BigBrain segmentation. The following transformations were applied using the TorchIO[1] package to the segmented BigBrain volume prior to extracting 2D sections.

1. Affine transformations: scale ~ uniform(0.9,1.1), rotations ~ uniform (0,15)

The following transformations were applied to the 2D synthetic images:

A. Random affine transforms
B. Scaling of 50% of images by a random exponent drawn: *exponent ~ gamme(k=2, Θ=1)*
C. Gaussian smoothing: $\sigma_{smoothing}$ ~ |*gaussian(μ=0, σ=1)*|
D. Gaussian noise: σ_{noise} ~ |*gaussian(μ=0, σ=0.01)*|
E. Masking out a random rectangle in 50% of images: *rectangle size = image dimensions x uniform(0, 0.2)*
F. Changing the zoom factor of the image by 80-120% : zoom ~ uniform(0.8, 1.2)
G. The application of a random bias field on 20% of synthetic volumes.
H. Random spike fields on 10% of synthetic volumes.
I. Random ghosting on 10% of synthetic volumes.

5.3 Parameters for antsRegistration

(1) Within the ANTs software package, “antsRegistration” is the software that calculates the optimal transformation between two images for a given kind of transformation.

5.3.1 Parameters for the initial inter-section alignment

(1) The rigid alignment described in Section 4.2.2 is performed hierarchically with ANTs at voxel sizes of 4.096mm, 1.024mm, and 0.256mm with smoothing kernels of 2mm, 0.5mm, and 0.125mm full width at half-maximum (FWHM), respectively, and 100, 50, and 25 iterations[2].

5.3.2 Parameters for alignment of the reconstructed volume to the structural reference volume

(1) The ANTs alignment parameters used in the 3D alignment of the reconstructed volume (4.2.3.2) and 2D alignment of the individual sections (4.2.3.3) were set as follows. First, the transformations were calculated in the following order: rigid (parameters: 3 translations + 3 rotations), similarity transform (parameters: rigid + 1 global scaling), affine (parameters: rigid parameters + 3 scaling + 3 sheering parameters), SyN non-linear transformation with the Mattes mutual information (MI)[3] metric, and SyN non-linear transformation with local cross-correlation (LCC). The rigid transformation was initialized by antsRegistration using the center of mass of the fixed and moving moving images. Both images were GM segmentations, hence the center

of mass should be the center of the cortex. Subsequent transformations were initialized using the previous transformation in the series.

(2) For each alignment step in the multi-resolution hierarchy, the downsample factors passed to antsRegistration were set such that the alignment would be calculated using all the previous resolutions in the resolution hierarchy. That is, the pipeline is currently at resolution 1.0mm out of a hierarchy consisting of *r = (4.0mm, 3.0mm, 2.0mm, 1.0mm, 0.5mm)*, then antsRegistration will be set so that it calculates the optimal transformation between the specified images at 4.0mm, 3.0mm, 2.0mm, and finally the current resolution of 1.0mm. Hence the downsample factors, d_i, are calculated as

$$d_i = r_i / r_c$$

where i is an integer $i \in \{0,1,...,c\}$ that indexes the resolution hierarchy, r, from the lowest level resolution in the hierarchy, r_0=4.0mm, to the current level, r_c=1.0mm.

(3) The smoothing factors, s_i, were calculated by:

$$s_i = 0.2 \times d_i \text{ if } i < c, \text{ otherwise } 0$$

The smoothing factor s_c is equal to 0 because no smoothing needs to be performed for a downsample factor of 1 where images are at the current resolution, r_c. The scalar value of 0.2 is used because this is the equation used in the ANTs package to calculate smoothing factors based on downsampling factors.

(4) The number of iterations for each use of antsRegistration was calculated by multiplying a given number of iterations:

$$t_i = B_K \times (N - i),$$

where B_k is the base number of iterations for the kind of transformation $k \in \{$linear, SyN with Mattes[3] MI, SyN with LCC$\}$, N is the the number of levels in multiresolution hierarchy r, i.e., here N=6, and i is, as above, an integer that indexes r. B_k equals 500 for linear transformation, 200 for SyN with Mattes[3] MI, and 100 for SyN with LCC.

(5) The distance metric is calculated by sampling 90% of the pixels or voxels in the image. For the 2D alignment, the linear alignment is calculated multiple times and the best alignment, as quantified by Dice score between the binary images, is used to initialize the non-linear alignment step. This is done because the linear alignment occasionally failed and the moving image would be severely misaligned. Heuristically, 5 attempts at linear alignment proved sufficient.

5.4 Cytoarchitectonic Areas

(1) A total of 29 cytoarchitectonic areas spanning all the lobes of the cortex were chosen for comparison between raw 2D autoradiographs and 3D reconstructed receptor volumes. All

available areas that were defined in both the manual 2D autoradiograph annotations[4] and the Julich Brain Atlas [5].

Visual (hOc4v)	PSC (1)
Visual (hOc4d)	PSC (3a)
Visual (hOc6)	PSC (3b)
Visual (hOc3d)	SPL (5M)
Visual (hOc1)	SPL (5L)
Visual (hOc2)	SPL (7M)
IPL (PFm)	OFC (Fo4)
IPL (PFt)	OFC (Fo3)
IPL (PFop)	OFC (Fo5)
IPL (PGp)	Cingulum (25)
Motor (4a)	Cingulum (s32)
Motor (4p)	Cingulum (p32)
Supplementary Motor (sma)	Cingulum (33)
Broca (44)	Cingulum (s24)
Broca (45)	

Table 2: List of cytoarchitectonic areas used to compare receptor densities in manually defined 2D regions in raw autoradiographs versus 3D regions in the reconstructed volumes defined on the Julich Brain atlas [5].

5.5 Validation Figures

Figure 2. Examples of synthetic data for training U-Net

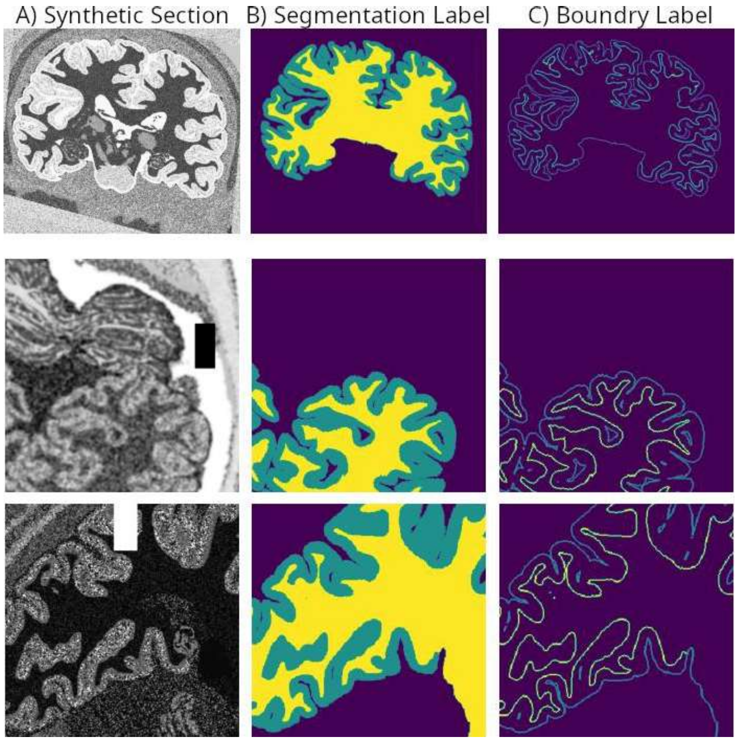

Example of generated synthetic images for training the segmentation U-Net. A) Examples are shown of the synthetic images with different random values assigned to cortical layers, WM, cerebrospinal fluid, skull, and background with added artefacts, including: i) affine transformation of the BigBrain volume, b) gaussian smoothing and noise, c) zoom factors, and d) occluded rectangular regions. Two sets of labels are used in the training: B) GM and WM regions, C) WM-GM and GM-pial surface boundaries.

Figure 3. Illustrative schema of surface-based interpolation algorithm.

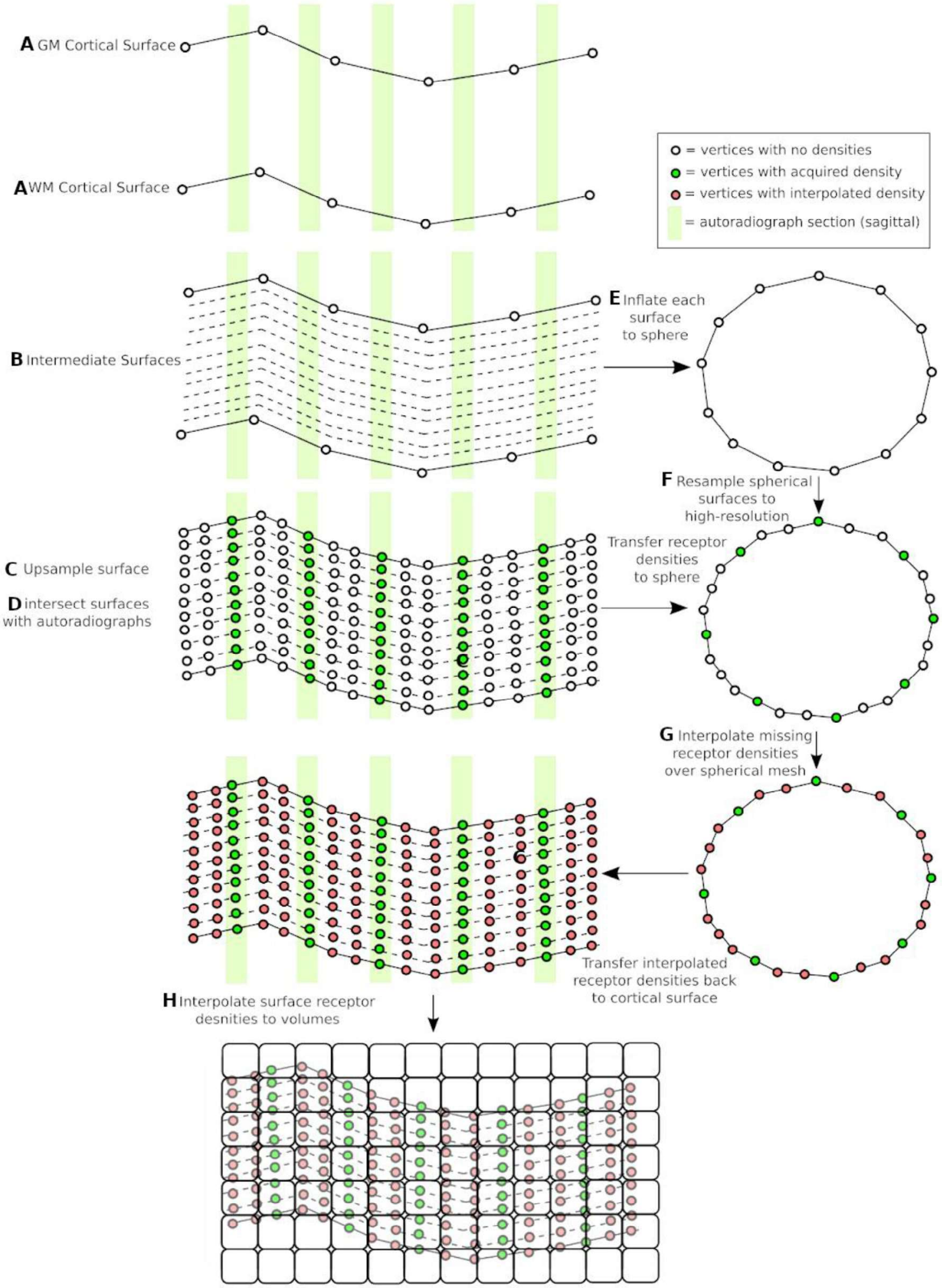
A GM Cortical Surface
A WM Cortical Surface
= vertices with no densities
= vertices with acquired density
= vertices with interpolated density
= autoradiograph section (sagittal)
B Intermediate Surfaces
E Inflate each surface to sphere
F Resample spherical surfaces to high-resolution
Transfer receptor densities to sphere
C Upsample surface
D intersect surfaces with autoradiographs
G Interpolate missing receptor densities over spherical mesh
Transfer interpolated receptor densities back to cortical surface
H Interpolate surface receptor desnities to volumes

Intermediate meshes are defined between the WM and GM surfaces. The cortical surfaces are upsampled and pixel intensities of autoradiographs are projected onto the surface (green circles). Each of the surfaces is inflated to a sphere. Missing pixel intensities are interpolated over the spherical surfaces (red circles). The acquired and interpolated pixel intensities are interpolated into a volume to produce a volumetric atlas of receptor density for a specific receptor.

Figure 4: Random parcellations used to validate interpolation error

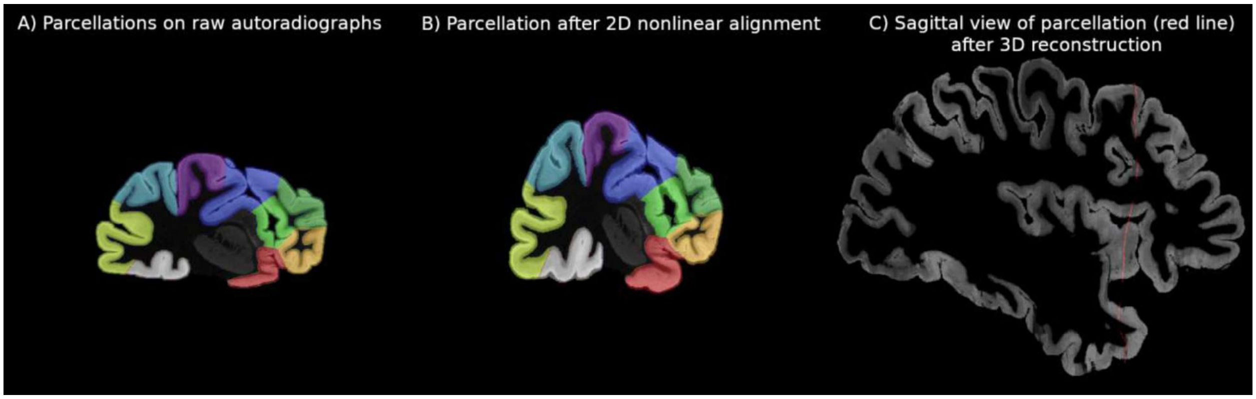

The regions of interest were transformed using the 2D non-linear transformation (B) and the 3D non-linear transformation (C) of the autoradiograph on which they were defined. After 3D transformation, the autoradiographs and corresponding parcellations are no longer defined in a 2D plane but in a 3D volume. Hence the right-most image shows the 3D reconstructed volume from a sagittal view with the red line showing the position of the warped autoradiograph and parcellation after 3D transformation.

References

1. TorchIO: A Python library for efficient loading, preprocessing, augmentation and patch-based sampling of medical images in deep learning. *Comput. Methods Programs Biomed.* **208**, 106236 (2021).
2. Tustison, N. J. *et al.* The ANTsX ecosystem for quantitative biological and medical imaging. *Sci. Rep.* **11**, 9068 (2021).
3. Mattes, D., Haynor, D. R., Vesselle, H., Lewellyn, T. K. & Eubank, W. Nonrigid multimodality image registration. in *Medical Imaging 2001: Image Processing* (eds. Sonka, M. & Hanson, K. M.) (SPIE, 2001). doi:10.1117/12.431046.
4. Zilles, K. *et al.* Architectonics of the human cerebral cortex and transmitter receptor

fingerprints: reconciling functional neuroanatomy and neurochemistry. *Eur. Neuropsychopharmacol.* **12**, 587–599 (2002).

5. Amunts, K., Mohlberg, H., Bludau, S. & Zilles, K. Julich-Brain: A 3D probabilistic atlas of the human brain's cytoarchitecture. *Science* **369**, 988–992 (2020).
